# Determinants of non-vaccination against seasonal influenza during pregnancy

**DOI:** 10.17269/s41997-024-00871-z

**Published:** 2024-04-01

**Authors:** David Guan, Nicolas L. Gilbert, Mireille Guay, Aubrey Maquiling, Jackie Kokaua, Isabelle Lévesque, Vanessa Poliquin

**Affiliations:** 1 Centre for Immunization Surveillance, Public Health Agency of Canada, Ottawa, Ontario Canada; 2 École de santé publique de l’Université de Montréal, Montréal, Québec Canada; 3 Centre for Population Health Data, Statistics Canada, Ottawa, Ontario Canada; 4 Department of Obstetrics, Gynecology, and Reproductive Sciences, Faculty of Health Sciences, University of Manitoba, Winnipeg, Manitoba Canada

**Keywords:** Pregnancy, Vaccination, Influenza, Canada, Grossesse, vaccination, grippe, Canada

## Abstract

**Objective:**

The objective of this study was to identify the determinants of influenza non-vaccination during pregnancy in Canada.

**Methods:**

Biological mothers of children born between December 2018 and March 2019 were surveyed about vaccinations they had received during pregnancy, reasons for non-vaccination, obstetrical history, and demographics. Simple and multiple logistic regression models were used to measure associations between various sociodemographic factors as well as obstetrical history, and non-vaccination against influenza. We analyzed data from 2361 mothers.

**Results:**

Factors associated with non-vaccination included being followed during pregnancy by a midwife compared to by an obstetrician-gynecologist (OR 2.02; 95% CI, 1.17‒3.50); having two or more past live births compared to none (OR 1.58; 95% CI, 1.01‒2.49); having an education level below high school diploma compared to a bachelor’s degree or above (OR 2.50; 95% CI, 1.06‒5.90); and having a household income below $60,000 (OR 2.46; 95% CI, 1.42‒4.24) or between $60,000 and $99,999 (OR 2.77; 95% CI, 1.70‒4.52) compared to a household income of $140,000 or more. The province or territory of prenatal care proved to be an important factor in non-vaccination, with statistically significant odds ratios for certain provinces: OR 7.50 (95% CI, 1.40‒40.26) for Ontario, 8.23 (95% CI, 1.53‒44.23) for Newfoundland and Labrador, and 11.39 (95% CI, 2.14‒60.60) for Quebec, as compared to the territories.

**Conclusion:**

Despite universal access to influenza vaccines in Canada during pregnancy, regional variations and socioeconomic disparities in non-vaccination are still observable.

## Résumé

**Objectif:**

Identifier les déterminants de la non-vaccination contre la grippe pendant la grossesse au Canada.

**Méthodes:**

Notre étude porte sur 2 361 mères biologiques d’enfants nés entre décembre 2018 et mars 2019 qui ont été interrogées sur les vaccins reçus pendant leur grossesse, les raisons de non-vaccination, leurs antécédents obstétricaux, et leurs caractéristiques démographiques. Des modèles de régression logistique simple et multiple ont été utilisés pour mesurer les associations entre divers facteurs sociodémographiques, les antécédents obstétricaux, et la non-vaccination contre l’influenza.

**Résultats:**

Les facteurs associés à la non-vaccination comprennent le suivi de grossesse par une sage-femme par rapport à un obstétricien-gynécologue (RC 2,02; IC 95% : 1,17‒3,50); avoir eu deux naissances vivantes ou plus par rapport à aucune (RC 1,58; IC 95% : 1,01‒2,49); avoir une scolarité inférieure au diplôme d’études secondaires par rapport à un baccalauréat ou plus (RC 2,50; IC 95% : 1,06‒5,90); et avoir un revenu du ménage inférieur à 60 000 $ (RC 2,46; IC 95% : 1,42‒4,24) ou entre 60 000 $ et 99 999 $ (RC 2,77; IC 95% : 1,70‒4,52) par rapport à un revenu ménager de 140 000 $ ou plus. La province ou le territoire de soins prénataux s’est avéré un facteur important de la non-vaccination avec des rapports de cote statistiquement significatifs pour certaines provinces : RC 7,50 (IC 95% : 1,40‒40,26) pour l’Ontario, 8,23 (IC 95% : 1,53‒44,23) pour Terre-Neuve-et-Labrador, et 11,39 (IC 95% : 2,14‒60,60) pour le Québec, comparativement aux territoires.

**Conclusion:**

Malgré l’accès universel aux vaccins antigrippaux au Canada durant la grossesse, des variations régionales et des disparités socioéconomiques en non-vaccination persistent.

## Introduction

Influenza is a significant cause of morbidity and mortality in Canada, where it causes on average 12,000 hospitalizations and 3500 deaths annually (PHAC, 2021). Pregnant persons and infants younger than 6 months are at a significantly increased risk of influenza-related hospitalizations (Dodds et al., 2007; Neuzil et al., 2000; Schanzer et al., 2007). The risk of influenza-related hospitalization increases with gestational age, being higher in the third trimester than in the second (Dodds et al., 2007). There is strong evidence that vaccination during pregnancy not only protects pregnant persons from risk of influenza complications, but is also effective at protecting their newborns from influenza and influenza-related hospitalization (Jarvis et al., 2020). There is also evidence suggesting that influenza vaccination during pregnancy may reduce the risk of adverse birth outcomes; however, the literature reporting on this outcome is conflicting (Fell et al., 2017). The influenza vaccine is safe: its administration during pregnancy is not associated with increased risks for stillbirth, birth defects, or any other adverse events for the mother or the fetus (Lu et al., 2021; Moro et al., 2011).

Since 2007, the National Advisory Committee on Immunization (NACI) recommends that seasonal influenza vaccine be offered at each pregnancy (NACI, 2007). Similarly, the Society of Obstetricians and Gynecologists of Canada (SOGC) recommends that influenza vaccine be offered at any gestational age when pregnancy overlaps the influenza season (Castillo & Poliquin, 2018).

Insufficient uptake of influenza vaccination during pregnancy in Canada is a significant concern as influenza can lead to serious consequences for both the mother’s and the child’s health. Despite recommendations from health authorities, many pregnant persons are not getting vaccinated. For the 2018–2019 influenza season, Nova Scotia reported that pregnant females had the lowest coverage rate (14.4%) among their target groups for influenza vaccination coverage (Department of Health & Wellness, 2019). The most recent estimate of seasonal influenza vaccination coverage during pregnancy for all of Canada was 53% in 2020–2021 (PHAC, 2022). This is an improvement compared to the previous national estimate of 45% but it remains suboptimal. Notably, the SOGC in Canada has undertaken initiatives to promote vaccination among pregnant persons, including the development of clinical practice guidelines specific to immunization during pregnancy and the provision of comprehensive educational resources (Green, 2020). Despite these efforts, the persistently suboptimal vaccination rates highlight the need for ongoing research into more effective strategies to enhance influenza vaccine uptake. A deeper understanding of the factors associated with non-vaccination could provide insights for designing targeted interventions and strategies to increase uptake among pregnant persons in Canada. Research attempting to elucidate this relationship is sparse and inconsistent in the Canadian context (Greyson et al., 2021; Poliquin et al., 2019).

This study aims to identify sociodemographic and obstetrical history factors associated with non-vaccination against influenza during pregnancy in Canada.

## Methods

### Population of study

The childhood National Immunization Coverage Survey (cNICS) is a cross-sectional survey conducted every 2 years by Statistics Canada in collaboration with the Public Health Agency of Canada since 2011 and its purpose is to collect information on national coverage for vaccines administered to children and adolescents. The survey is based on a complex sampling design allowing for a representative sample of the Canadian population.

For the first time, in 2019 the cNICS also included a component on prenatal vaccination called the Survey of Vaccination during Pregnancy whose data were used in these analyses. The target population of the survey consisted of all mothers who gave birth between September 2, 2018, and March 1, 2019, living in the 10 provinces and 3 territories, not residing on First Nations reserves, and not institutionalized (such as in long-term care facilities and prisons). The survey frame was built using the list of applicants to the Canada Child Benefit (CCB), which was estimated to include 96% of children in Canada in 2018. Collected data included information about the vaccines offered and received during the prenatal period; knowledge, attitudes, and beliefs regarding vaccination; and sociodemographic information. Data on vaccines received during pregnancy were collected via a self-response electronic questionnaire or a computer-assisted telephone interview between December 2019 and March 2020. A more detailed description of the survey’s data sources and methodology is available on Statistics Canada’s website (Statistics Canada, 2020).

In 2019, the 10 provinces and 3 territories began their influenza vaccination programs on different dates, with the latest, Quebec, having started on November 1. For this reason, among the 5091 biological mothers surveyed, only 2429 mothers who gave birth between December 2018 and March 2019, i.e., those who had at least 1 month to get vaccinated before the end of their pregnancy, were included in this analysis.

### Variables

The dependent variable was self-reported influenza vaccination status during the recent pregnancy of the biological mothers, determined by the response to the following question: “Did you receive the flu (influenza) vaccine during your pregnancy?” The independent variable concerning advice from a prenatal care provider to get vaccinated against influenza was determined by the question: “Did your [primary maternal care provider] advise you to get the flu (influenza) vaccine, during your pregnancy?” with response options being “Yes”, “No”, and “Don’t know”. Other independent variables included the type of primary prenatal care provider, number of past live births, marital status, highest education level of the mother, country of birth of the mother, Indigenous status, province of prenatal care, rural/urban indicator, household income, mother’s age at time of child’s birth, and the child’s month of birth. These variables were selected because they have been cited in the scientific literature as being potentially associated with non-vaccination (Irving et al., 2021; Okoli et al., 2021; Poliquin et al., 2019).

For the 2019 cycle of cNICS, income information was not collected. Household income was provided from administrative data sources and was imputed when missing (0.1% of records) using the nearest neighbour method (Rancourt, 1999). The numerical variables household income and mother’s age were recoded into quartiles. Household income quartile boundaries were rounded up to the nearest $10,000. Due to their low sample size, the three territories were grouped to increase statistical power and adhere to confidentiality policies. Additionally, respondents whose education level was “Trades certificate or diploma” were grouped with respondents who selected “College/CEGEP/other non-university certificate or diploma” and “University certificate or diploma below the bachelor’s level”. Finally, “Bachelor’s degree” responses were grouped with “University certificate, diploma, degree above the BA level”.

### Statistical analysis

Unless specified otherwise, calculated proportions and odds ratios (ORs) are weighted whereas the absolute counts are unweighted. To improve the representativeness, survey weights were used to compensate for the unequal selection probability and were adjusted for non-response. The 95% confidence intervals of the proportions were estimated using the modified Wilson method (Franco et al., 2019). Simple and multiple logistic regressions were fitted to test for the associations between the various factors and non-vaccination against influenza during pregnancy and unadjusted and adjusted odds ratios were computed, respectively. The confidence intervals for the odds ratios were adjusted for multiple comparisons using Dunnett’s method. All factors except advice to get vaccinated against influenza were included in the multiple logistic regression. As observed for pertussis in the same survey (Gilbert et al., 2022), being advised to get vaccinated against influenza was very strongly associated with the mother being vaccinated in simple logistic regression (Table 3). However, this variable was not included in the multiple regression because it was deemed to be an intermediate step in the causal pathway between other independent variables and non-vaccination. Multicollinearity was evaluated by conducting Rao-Scott likelihood ratio chi-square tests to examine associations among all independent variables. Although certain variables showed associations, there were no indications of severe multicollinearity such as excessively large standard errors. The measures of variance (95% confidence intervals and standard errors) were estimated using the bootstrap method to account for the complex sampling design. SAS 9.4 was used to perform the statistical analyses; *p* < 0.05 indicated statistical significance.

## Results

### Sample characteristics

Overall, 5091 mothers answered the survey out of the initial sample. After adjusting for out-of-scope units, the overall unweighted response rate was 58.9% at the national level. The response rate by province and territory ranged from 30.9% to 67.1%. Among our subsample of 2429 respondents, 68 respondents (2.8%) did not give a response to the question, “Did you receive the flu (influenza) vaccine during your pregnancy?” which was used to create the dependent variable (non-vaccination) and as such, they were excluded from the analyses. Thus, the final sample for analysis comprises 2361 respondents. Characteristics of the final sample can be found in Table 1. The proportion of non-vaccinated in the final sample was 55.0% (95% CI, 52.1‒57.9).

**Table 1:** Characteristics of the study population

Variable	*n*^a^	Unweighted %	Weighted % (95% CI)
Primary prenatal care provider^b^			
Obstetrician-gynecologist	1377	58.4	61.9 (59.1–64.5)
General practitioner (family doctor)	661	28.0	22.0 (19.9–24.3)
Midwife	215	9.1	13.0 (11.1–15.1)
Nurse	NR	NR	NR
Other healthcare provider/no prenatal care	NR	NR	NR
Number of past live births			
None	1052	44.6	43.7 (40.7–46.8)
1	864	36.6	36.0 (33.1–39.1)
2 +	443	18.8	20.2 (17.9–22.8)
Marital status			
Married/common law	2105	89.2	85.6 (83.2–87.7)
Never married/separated/divorced/widowed	250	10.6	14.4 (12.3–16.8)
Education			
Below high school diploma	103	4.4	5.0 (3.8–6.6)
High school diploma	401	17.0	16.8 (14.7–19.1)
Post-secondary education below bachelor’s (including trade school)	787	33.3	34.9 (32.1–37.9)
Bachelor’s or above	1060	44.9	43.3 (40.5–46.2)
Mother’s country of birth			
Canada	1812	76.7	69.3 (66.5–72.0)
Outside of Canada	548	23.2	30.7 (28.0–33.5)
Indigenous status			
Not Indigenous	2213	93.7	95.4 (94.1–96.4)
Indigenous	144	6.1	4.6 (3.6–5.9)
Province of prenatal care			
Newfoundland and Labrador	203	8.6	1.2 (0.8–1.7)
Prince Edward Island	148	6.3	0.4 (0.2–0.7)
Nova Scotia	202	8.6	2.0 (1.5–2.7)
New Brunswick	222	9.4	1.9 (1.4–2.5)
Quebec	270	11.4	22.6 (20.9–24.5)
Ontario	252	10.7	35.7 (33.4–38.2)
Manitoba	195	8.3	4.1 (3.4–5.0)
Saskatchewan	215	9.1	4.1 (3.4–5.0)
Alberta	291	12.3	15.1 (13.7–16.6)
British Columbia	253	10.7	12.1 (10.9–13.5)
Territories	110	4.7	0.7 (0.4–1.2)
Rural/urban indicator			
Rural	561	23.8	16.0 (14.1–18.2)
Urban	1774	75.1	84.0 (81.8–85.9)
Household income			
0–$59,999	621	26.3	32.7 (30.0–35.5)
$60,000–$99,999	599	25.4	24.7 (22.4–27.1)
$100,000–$139,999	517	21.9	19.9 (17.8–22.2)
$140,000 or more	624	26.4	22.7 (20.6–24.9)
Mother’s age			
15–28	711	30.1	29.1 (26.3–32.1)
29–31	563	23.8	21.8 (19.6–24.2)
32–34	507	21.5	22.5 (20.0–25.2)
35–52	555	23.5	26.6 (23.9–29.4)
Child’s month of birth			
December	796	33.7	32.7 (30.0–35.4)
January	795	33.7	34.0 (31.3–36.8)
February or March	770	32.6	33.4 (30.7–36.2)
Advised to get vaccinated against influenza			
Yes	1583	66.3	66.3 (63.3–69.3)
No	622	33.7	33.7 (30.7–36.7)
Vaccinated against influenza during pregnancy			
Yes	1310	55.5	45.0 (42.1–47.9)
No	1051	44.5	55.0 (52.1–57.9)

*n* values are unweighted

^a^*n* = 2361; however, columns may not add up to it due to missing data

^b^May or may not be the healthcare provider who attended the child’s birth

*NR*, not reportable. Some estimates and their associated confidence intervals are marked as “NR”. This is due to their statistical quality not meeting the necessary standards. They exhibit high instability which renders them potentially misleading and unreliable. Drawing conclusions based on these data may lead to invalid results

### Reasons for non-vaccination

Among the 1051 respondents who were not vaccinated against influenza during pregnancy, the reasons most commonly cited were the following: “I did not want to get the flu vaccine while I was pregnant” (46.1%) and “I was not aware that the flu vaccine was recommended during pregnancy” (23.0%) (Table 2). Some cited reasons for non-vaccination related to other aspects of accessibility such as “It would have been necessary to visit a different healthcare provider and/or the flu vaccine was not offered by my primary healthcare provider” (7.3%).

**Table 2:** Top reasons declared by mothers for not getting vaccinated against influenza during pregnancy

Reason	% (95% CI)^a^
I did not want to get the flu vaccine while I was pregnant	46.1 (41.8‒50.6)
I was not aware that the flu vaccine was recommended during pregnancy	23.0 (19.6‒26.8)
The flu vaccine would not have protected me against the flu	6.0 (4.2‒8.4)
The flu vaccine was not recommended by my primary healthcare provider	5.7 (4.0‒7.9)
The flu vaccine was not offered by my primary healthcare provider and/or it would have been necessary to visit a different healthcare provider to get the vaccine	7.3 (5.5‒9.6)
The flu vaccine could have been harmful for my baby	4.9 (3.5‒6.8)

Percentages are weighted

^a^Respondents could answer multiple reasons so total could exceed 100%. *n* = 1051 respondents who chose not to get vaccinated against influenza. Missing responses (< 1%) were excluded

### Sociodemographic factors and obstetrical history

In simple regressions, lower education level, province of care, lower income, a younger mother’s age, two or more past live births, and not having been advised to get the vaccine were factors significantly associated with non-vaccination (Table 3). In simple logistic regression, not being advised by the prenatal care provider to get vaccinated had the strongest association with non-vaccination (OR 12.91; 95% CI, 8.99‒18.54). After controlling for other variables, the mother’s age was no longer significantly associated with influenza non-vaccination in the multiple regression model. There were no significant differences among mothers who received prenatal care from obstetrician-gynecologists, family doctors, nurses/other prenatal care providers, or midwives in terms of receiving advice to get vaccinated against influenza during pregnancy (Table 4).

**Table 3:** Determinants of non-vaccination against influenza during pregnancy

Variable	*n*	% unvaccinated (95% CI)	Unadjusted OR (95% CI)	Adjusted OR^a^ (95% CI)
Primary prenatal care provider^b^				
Obstetrician-gynecologist	1341	53.7 (49.8‒57.5)	Reference	Reference
General practitioner (family doctor)	643	53.5 (47.8‒59.1)	0.99 (0.70‒1.41)	1.04 (0.70‒1.55)
Midwife	212	64.0 (55.8‒71.4)	1.53 (0.94‒2.51)	2.02 (1.17‒3.50)*
Nurse	NR	NR	NR	NR
Other healthcare provider/no prenatal care	NR	NR	NR	NR
Number of past live births				
None	1024	51.7 (47.2‒56.2)	Reference	Reference
1	847	53.3 (48.4‒58.1)	1.07 (0.79‒1.44)	1.12 (0.81‒1.56)
2 +	426	65.3 (58.7–71.3)	1.76 (1.19–2.59)*	1.58 (1.01–2.49)*
Marital status				
Married/common law	2052	53.5 (50.5–56.6)	Reference	Reference
Never married/separated/divorced/widowed	245	63.1 (54.0–71.3)	1.48 (0.99–2.22)	0.92 (0.55–1.54)
Education				
Below high school diploma	101	75.3 (64.2–83.8)^c^	3.71 (1.80–7.64)*^c^	2.50 (1.06–5.90)*^c^
High school diploma	392	64.0 (56.9–70.5)	2.17 (1.41–3.33)*	1.47 (0.87–2.49)
Post-secondary education below bachelor’s (including trade school)	771	59.7 (54.8–64.5)	1.81 (1.30–2.52)*	1.29 (0.88–1.88)
Bachelor’s or above	1033	45.0 (40.7–49.5)	Reference	Reference
Mother’s country of birth				
Canada	1773	53.0 (49.5–56.5)	Reference	Reference
Outside of Canada	524	59.4 (54.0–64.6)	1.30 (1.00–1.69)	1.15 (0.82–1.60)
Indigenous status				
Not Indigenous	2157	54.5 (51.4–57.5)	Reference	Reference
Indigenous	140	65.0 (53.3–75.1)	1.55 (0.92–2.63)	1.44 (0.78–2.66)
Province of prenatal care				
Newfoundland and Labrador	201	58.6 (51.6–65.3)	4.79 (1.56–14.68)*	8.23 (1.53–44.23)*
Prince Edward Island	147	24.8 (18.4–32.4)	1.11 (0.35–3.51)	1.55 (0.28–8.55)
Nova Scotia	197	28.5 (22.0–36.1)	1.35 (0.42–4.27)	1.77 (0.32–9.88)
New Brunswick	215	41.6 (33.7–50.1)	2.41 (0.76–7.60)	3.39 (0.63–18.13)
Quebec	266	67.1 (61.3–72.4)	6.89 (2.27–20.96)*	11.39 (2.14–60.60)*
Ontario	241	56.7 (50.4–62.8)	4.42 (1.44–13.54)*	7.50 (1.40–40.26)*
Manitoba	187	51.2 (43.6–58.8)	3.55 (1.16–10.85)*	4.44 (0.84–23.55)
Saskatchewan	203	35.1 (27.9–43.0)	1.83 (0.58–5.79)	2.29 (0.41–12.74)
Alberta	285	50.6 (44.7–56.5)	3.46 (1.14–10.52)*	5.31 (1.00–28.25)
British Columbia	250	50.2 (43.8–56.6)	3.41 (1.12–10.35)*	5.23 (0.96–28.49)
Territories	105	22.8 (10.8–42.0)	Reference	Reference
Rural/urban indicator				
Rural	551	56.5 (49.7–63.0)	1.08 (0.80–1.45)	1.04 (0.73–1.49)
Urban	1746	54.7 (51.5–57.8)	Reference	Reference
Household income				
0–$59,999	601	64.3 (58.3–69.8)	3.19 (2.09–4.86)*	2.46 (1.42–4.24)*
$60,000–$99,999	583	65.4 (60.1–70.4)	3.35 (2.24–5.01)*	2.77 (1.70–4.52)*
$100,000–$139,999	507	48.7 (42.4–55.0)	1.68 (1.10–2.57)*	1.51 (0.95–2.38)
$140,000 or more	606	36.1 (30.8–41.7)	Reference	Reference
Mother’s age				
15–28	700	63.5 (58.0–68.8)	1.59 (1.08–2.35)*	1.44 (0.89–2.33)
29–31	551	48.4 (42.5–54.4)	0.86 (0.57–1.29)	1.04 (0.66–1.64)
32–34	498	52.7 (46.3–59.0)	1.02 (0.66–1.56)	1.02 (0.64–1.63)
35–52	548	52.3 (46.6–57.9)	Reference	Reference
Child’s month of birth				
December	773	59.4 (54.5–64.1)	1.36 (0.99–1.88)	1.34 (0.95–1.90)
January	776	54.1 (48.9–59.1)	1.10 (0.80–1.52)	1.11 (0.76–1.60)
February or March	748	51.7 (46.8–56.6)	Reference	Reference
Advised to get vaccinated against influenza				
Yes	1582	35.7 (32.2–39.4)	Reference	
No	621	87.8 (84.0–90.8)	12.91 (8.99–18.54)*	

*n* values are unweighted. Percentages and odds ratios are weighted. Missing data for all variables were excluded in the simple and multiple regression models. Sample size for the multiple regression model was *n* = 2297

^a^Adjusted for all the other variables present in this column

^b^May or may not be the professional who attended the child’s birth

^c^Estimates and confidence intervals are considered to be of marginal quality due to high sampling variability, and should be used with caution

*NR*, not reportable. Some estimates and their associated confidence intervals are marked as “NR”. This is due to their statistical quality not meeting the necessary standards. They exhibit high instability which renders them potentially misleading and unreliable. Drawing conclusions based on these data may lead to invalid results

*Statistically significant (*p* < 0.05)

**Table 4:** Association between prenatal care provider and lack of advice to get vaccinated against influenza during pregnancy

Variable	*n*	% not advised to vaccinate^a^ (95% CI)	Unadjusted OR^a^ (95% CI)
Primary prenatal care provider^b^			
Obstetrician-gynecologist	1293	33.0 (29.2‒37.0)	0.98 (0.56‒1.70)
General practitioner (family doctor)	629	35.9 (30.3‒41.8)	1.11 (0.62‒2.00)
Midwife	187	33.5 (25.4‒42.7)	Reference
Nurse	NR	NR	NR
Other healthcare provider	NR	NR	NR

*n* values are unweighted. Percentages and odds ratios are weighted

^a^Mothers who were not advised by their prenatal care provider to get vaccinated against influenza during pregnancy. This does not mean that they were advised against vaccination

^b^May or may not be the healthcare provider who attended the child’s birth. Mothers who received no prenatal care at all or who did not remember/state whether they received advice were excluded from this analysis. Sample size for this analysis was *n* = 2203

*NR*, not reportable. Some estimates and their associated confidence intervals are marked as “NR”. This is due to their statistical quality not meeting the necessary standards. They exhibit high instability which renders them potentially misleading and unreliable. Drawing conclusions based on these data may lead to invalid results

Factors independently associated with non-vaccination included having an educational level below high school diploma (OR 2.50; 95% CI, 1.06–5.90) compared to a bachelor’s degree or above; and having a household income below $60,000 (OR 2.46; 95% CI, 1.42‒4.24) or between $60,000 and $99,999 (OR 2.77; 95% CI, 1.70‒4.52) compared to a household income of $140,000 or more. Being followed by a midwife during pregnancy was also associated with non-vaccination compared to being followed by an obstetrician-gynecologist (OR 2.02; 95% CI, 1.17‒3.50). Similarly, having two or more past live births was associated with non-vaccination compared to having none (OR 1.58; 95% CI, 1.01‒2.49).

The proportion of non-vaccinated pregnant persons varied significantly among the provinces and territories. For instance, in the territories (Northwest Territories, Yukon, and Nunavut), 22.8% (95% CI, 10.8‒42.0) were not vaccinated against influenza during pregnancy, whereas in Quebec, it was 67.1% (95% CI, 61.3‒72.4). Compared to the territories, receiving prenatal care in Quebec exhibited the strongest association with non-vaccination (OR 11.39; 95% CI, 2.14‒60.60), followed by Newfoundland and Labrador (OR 8.23; 95% CI, 1.53‒44.23), and Ontario (OR 7.50; 95% CI, 1.40‒40.26).

## Discussion

This survey examined the associations between socioeconomic factors as well as obstetrical history, and non-vaccination against influenza during pregnancy. The findings of this study suggest that even though influenza vaccination during pregnancy is widely available and publicly funded in Canada, there are still regional and socioeconomic disparities in non-vaccination. Specifically, those receiving prenatal care in Quebec are less likely to receive the influenza vaccine during their pregnancy than those in some other provinces and territories. Unlike other Canadian provinces and territories that recommend vaccination at any stage of pregnancy for healthy pregnant persons, the Quebec Immunization Committee advises vaccination during the second and third trimesters (Comité sur l’immunisation du Québec, 2018). This policy, based on their research indicating limited data for first-trimester vaccination and comparable influenza risk with non-pregnant persons, may influence perceptions of vaccine safety and uptake among pregnant persons in Quebec. Further research is needed to understand these significant variations in non-vaccination across Canada.

The results of this study show that there are socioeconomic inequalities in the uptake of influenza vaccination during pregnancy, with lower household income being associated with non-vaccination. However, there was not a significant difference between those with a household income of less than $59,999 and those with a household income of $60,000 to $99,999. Of the 1051 unvaccinated pregnant persons, less than 8% mentioned problems accessing the influenza vaccine as their reason for non-vaccination, suggesting that accessibility issues are unlikely to be the main cause. Additionally, a lower level of education was found to be a determinant for non-vaccination. It is important to note that there may be a correlation between education and household income, as both are indicators of socioeconomic status (SES). It is also possible that those with a lower SES may be less likely to adopt preventive health measures like vaccination due to lower literacy levels. A systematic review found that “locally designed, multicomponent interventions have evidence of effectiveness in urban, ethnically diverse, deprived populations” (Crocker-Buque et al., 2017). While this review addresses childhood vaccinations, similar strategies could potentially be effective for maternal vaccinations in comparable settings.

The results of this study also suggest that the risk of non-vaccination varied depending on the primary prenatal care providers, as pregnant persons receiving care from midwives were significantly less likely to get vaccinated against influenza than those receiving care from family physicians or obstetrician-gynecologists. Comparison with other Canadian studies on this topic reveals some similarities and differences.

For instance, Dubé et al. (2020) documented that persons with a family physician as their prenatal care provider are more likely to receive the influenza vaccine than those followed by a midwife during pregnancy, potentially due to knowledge gaps about the influenza vaccine among Canadian health professionals. This study found that midwives had a statistically significant lower level of knowledge and confidence in discussing vaccination with pregnant patients than family physicians and nurses. Additionally, family physicians and nurses also had more favourable views of vaccination during pregnancy, while midwives had more reservations. Prenatal care providers who had more positive attitudes towards vaccination were more likely to recommend it to pregnant patients, but about half of the prenatal care providers did not offer vaccination services in their practice because they did not feel equipped to do so or thought it was not their role. For example, midwives in Quebec or Ontario are not legally allowed to administer the influenza vaccine themselves. In a separate study, Dubé et al. (2013) also reported that physicians may use a more persuasive approach when discussing vaccination with parents, while midwives adopt a more neutral stance and provide information on the pros and cons of vaccination, leaving the decision up to the parents and promoting the principle of informed choice. On the contrary, our study indicates that there is no statistical discrepancy in vaccination recommendation between midwives and other prenatal care providers. This suggests that the higher non-vaccination observed among midwives’ patients may not be simply attributed to midwives’ knowledge, attitude, or model of care. It appears plausible that the association between non-vaccination for influenza during pregnancy and prenatal care by a midwife stems from the fact that persons who elect to be followed by midwives possess distinct views on this subject from the outset. Nevertheless, since midwives not addressing clients’ vaccine concerns might represent a missed opportunity to positively influence those views, it is vital to provide midwives with the knowledge and resources they need to offer a fully informed choice about vaccines. According to Pringle et al. (2022), successful informed choice discussions involve clients gaining full knowledge of vaccination interventions and making decisions, regardless of whether they follow the midwife’s recommendation. However, the same study describes that Canadian midwives often face challenges due to limited confidence in vaccine knowledge and counselling skills, highlighting the need for additional support tailored to the midwifery model of care.

On the other hand, unlike some studies (Dubé et al., 2020; Hilderman et al., 2011), our results do not show a statistical difference in vaccination uptake between patients followed by an obstetrician-gynecologist and those followed by a family physician. This could be due to the now more widespread knowledge that vaccination during pregnancy is safe, as the Society of Obstetricians and Gynecologists of Canada recommends since 2018 that tetanus, diphtheria, and acellular pertussis (Tdap) vaccine be administered in every pregnancy between the 21st and 32nd weeks (Castillo & Poliquin, 2018). A Canadian study found that obstetrician-gynecologists were more likely to support vaccination in pregnancy than family physicians, but less likely to offer it because they believed it was the responsibility of family physicians or local public health agencies to provide vaccination (Tong et al., 2008).

Our study identified an association between multiparity (having had two or more past live births) and non-vaccination during pregnancy, compared to nulliparity. The precise reasons for this association remain unclear, but several factors could potentially contribute. For instance, multiparous persons, who have experienced pregnancy before, are less likely to seek prenatal healthcare (Feijen-de Jong et al., 2012). Our analysis suggests that lack of advice from a prenatal care provider is strongly associated with non-vaccination, which could reduce the likelihood of these individuals receiving the influenza vaccine.

Consistent with previous research, our results show that household income and education are associated with vaccination uptake among pregnant persons (Poliquin et al., 2019). However, we did not find a significant association between non-vaccination against influenza during pregnancy and immigration status, urban–rural status, or age of the pregnant individual. A survey analysis by Greyson et al. (2021) found that demographics, such as income or education, and parity were not significant predictors of intentions to get vaccinated or vaccine uptake. This discrepancy may be because the participants in their study were asked about their intention to receive the influenza vaccine at the start of the influenza season, but they may have changed their minds during their pregnancy, possibly due to a recommendation from their prenatal care provider. Their second follow-up survey at the end of the influenza season had a participation rate of only 14%.

The results of our analysis differ from previous international studies. Okoli et al. (2021) conducted a systematic review and meta-analysis of global literature examining the sociodemographic and health-related determinants of seasonal influenza vaccination in pregnancy. Their study reported that maternal older age, being married, and living in a rural area were associated with increased seasonal influenza vaccine uptake, while our study found no such associations. The variation in the findings between this study and others conducted in other countries may be attributed to the distinctions in healthcare systems, cultural and societal factors, population characteristics, availability of the influenza vaccine, the design of the study, and the methods used for data collection. Furthermore, in contrast to our study, Irving et al. (2021) reported a statistically significant association between one or more past live births and increased influenza vaccination during pregnancy, although this relationship varied by country and across influenza seasons.

While our data predates the COVID-19 pandemic, its significance endures for several reasons. First, this dataset offers a crucial historical perspective, illuminating influenza vaccination practices during pregnancy before the intensified global spotlight on immunization. This benchmark is invaluable for gauging shifts in vaccination attitudes and behaviours in the aftermath of the pandemic. Moreover, our results highlight enduring socioeconomic disparities in non-vaccination. These persistent gaps, entrenched in deep-seated societal structures, are likely to prevail even amid a swiftly evolving health landscape. By identifying these patterns, we provide policymakers and public health professionals with insights crucial for addressing these ongoing disparities.

Further research is necessary to better understand the various factors influencing non-vaccination against influenza during pregnancy in Canada. For example, other barriers or facilitators frequently cited in the global literature, such as ethnicity, should be explored to determine whether cultural elements have an impact on non-vaccination in Canada. This analysis was not possible in our study because ethnicity was not collected as part of the survey. Additionally, future studies would benefit from improving immunization records across the country to provide more comprehensive and accurate estimates of vaccine uptake, which would help reduce non-response and recall biases.

### Strengths and limitations

A key strength of this research study is the survey design, which allowed for the selection of a representative sample of the population of pregnant persons in Canada. Moreover, the unweighted response rate was 58.9% at the national level, which is high enough to reduce non-response bias (Fincham, 2008).

A major limitation of this study is the small sample size for some subgroups, such as the individual territories or those persons followed by nurses during pregnancy. This made the resulting estimates potentially unreliable, limiting the conclusions that could be drawn. Moreover, the study excluded individuals residing on First Nations reserves, potentially overlooking the unique health determinants and experiences of Indigenous populations. Future research with a similar sample design could benefit from minimum quotas for specific groups to ensure that the sample is large enough for granular analyses. Another limitation is that the influenza vaccination status was self-reported and not confirmed by official immunization records, which may have resulted in a recall bias and possibly a social desirability bias. Nonetheless, the popularity of the influenza vaccine and the survey’s timing (about 1 year after birth) may have reduced the risk of recall bias.

## Conclusion

Even with universal access to influenza vaccine during pregnancy in Canada, significant socioeconomic inequalities in non-vaccination remain, notably among those with lower educational and income levels, and those under the care of midwives during pregnancy. Geographically, Ontario, Newfoundland and Labrador, and Quebec have markedly higher risk of non-vaccination compared to the territories. To more effectively guide health promotion activities, further investigation is needed to comprehend the reasons behind these inequalities and regional variations.

## Contributions to knowledge

What does this study add to existing knowledge?

- Factors independently associated with influenza non-vaccination during pregnancy were lower household income, lower education, multiparity, receiving prenatal care in certain provinces, or receiving prenatal care from a midwife as opposed to from a family doctor or an obstetrician-gynecologist.
- Few respondents reported that accessibility issues were the reason they did not get vaccinated against influenza during pregnancy.
- In simple logistic regression, not being advised to get vaccinated by the prenatal care provider was the most significant determinant of non-vaccination.

What are the key implications for public health interventions, practice, or policy?

- Public health interventions may need to target pregnant persons with lower household income and less education to increase awareness about the importance of influenza vaccination during pregnancy.
- Interventions may also need to consider working with all types of healthcare providers to ensure that pregnant persons receive counselling and education about influenza vaccination in line with public health recommendations.
- This study revealed inequalities in influenza vaccination during pregnancy but cannot fully explain them. To better inform decision-makers, further studies are needed to understand the mechanisms for overcoming the barriers to influenza vaccination.

## Data Availability

The data that support the findings of this study are accessible through Statistics Canada with restrictions.

## Code availability

The SAS codes used to analyze these data can be made available upon request.
